# Comparing findings from the random‐intercept cross‐lagged panel model and the monozygotic twin difference cross‐lagged panel model: Maladaptive parenting and offspring emotional and behavioural problems

**DOI:** 10.1002/jcv2.12203

**Published:** 2023-10-28

**Authors:** Marie‐Louise J. Kullberg, Charlotte C. Van Schie, Andrea G. Allegrini, Yasmin Ahmadzadeh, Daniel L. Wechsler, Bernet M. Elzinga, Tom A. McAdams

**Affiliations:** ^1^ Institute of Clinical Psychology Leiden University Leiden The Netherlands; ^2^ Leiden Institute for Brain and Cognition (LIBC) Leiden University Medical Centre Leiden The Netherlands; ^3^ School of Psychology and Illawarra Health and Medical Research Institute University of Wollongong Wollongong Australia; ^4^ Psychology and Language Sciences University College London London UK; ^5^ Social, Genetic and Developmental Psychiatry Centre Institute of Psychiatry, Psychology & Neuroscience King's College London London UK; ^6^ Promenta Research Centre University of Oslo Oslo Norway

**Keywords:** behavioural problems, causal inference, emotional problems, longitudinal methodology, maladaptive parenting

## Abstract

**Background:**

In this study we compare results obtained when applying the monozygotic twin difference cross‐lagged panel model (MZD‐CLPM) and a random intercept cross‐lagged panel model (RI‐CLPM) to the same data. Each of these models is designed to strengthen researchers' ability to draw causal inference from cross‐lagged associations. We explore differences and similarities in how each model does this, and in the results each model produces. Specifically, we examine associations between maladaptive parenting and child emotional and behavioural problems in identical twins aged 9, 12 and 16.

**Method:**

Child reports of 5698 identical twins from the Twins Early Development Study (TEDS) were analysed. We ran a regular CLPM to anchor our findings within the current literature, then applied the MZD‐CLPM and the RI‐CLPM.

**Results:**

The RI‐CLPM and MZD‐CLPM each enable researchers to evaluate the direction of effects between correlated variables, after accounting for unmeasured sources of potential confounding. Our interpretation of these models therefore focusses primarily on the magnitude and significance of cross‐lagged associations. In both the MZD‐CLPM and the RI‐CLPM behavioural problems at age 9 resulted in higher levels of maladaptive parenting at age 12. Other effects were not consistently significant across the two models, although the majority of estimates pointed in the same direction.

**Conclusion:**

In light of the triangulated methods, differences in the results obtained using the MZD‐CLPM and the RI‐CLPM underline the importance of careful consideration of what sources of unmeasured confounding different models control for and that nuance is required when interpreting findings using such models. We provide an overview of what the CLPM, RI‐CLPM and MZD‐CLPM can and cannot control for in this respect and the conclusions that can be drawn from each model.

## Graphical abstract

The random‐intercept cross‐lagged panel model (RI‐CLPM) and the monozygotic twin difference cross‐lagged panel model (MZD‐CLPM) are intended to improve the ability of researchers to draw causal inference. In both the MZD‐CLPM and the RI‐CLPM behavioural problems at age 9 resulted in higher levels of maladaptive parenting at age 12. In light of the triangulated methods, differences in the results obtained using the MZD‐CLPM and the RI‐CLPM underline the importance of careful consideration of what sources of unmeasured confounding different models control for and that nuance is required when interpreting findings using such models.

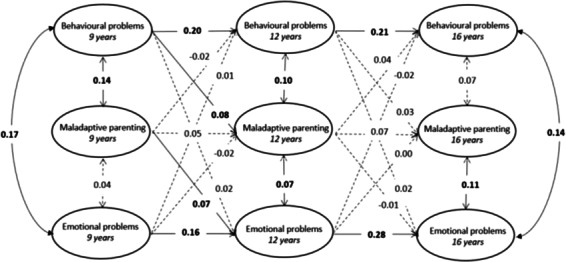

**Key Points**

- The random‐intercept cross‐lagged panel model and the monozygotic twin difference cross‐lagged panel model are intended to improve the ability of researchers to draw causal inference.
- Findings from both models indicated that child behavioural problems at age 9 predicted experienced maladaptive parenting at age 12.
- The results can be interpreted as corroborating (albeit not conclusive) evidence in favour of a causal relationship. However, most results differed across models.
- The substantial differences in results underline that (1) nuance is required when interpreting findings using such models and that (2) triangulating results across multiple (longitudinal) methods strengthens the ability to draw conclusions on causality.

## BACKGROUND

One of the key aims of developmental research is to identify factors that may causally influence child development. Extensive research has shown that an important predictor for the development of child emotional and behavioural problems is maladaptive parenting behaviour, including physical and verbal punishment and parenting strategies involving unclear and inconsistent communication with the child (see Baumrind, 1991; Crosnoe & Cavanagh, 2010; Fletcher et al., 2004). Equally, some studies have demonstrated that effects operate in the reverse direction: Child emotional and behavioural problems might elicit certain parenting practices (Pinquart, 2017a, 2017b). Understanding the nature and direction of associations between parenting behaviour and child problems is likely to prove important for prevention and intervention strategies to support families in fostering child mental well‐being. In order to understand the nature and direction of associations between variables, researchers often use longitudinal data. The direction of effects between parenting and child emotional and behavioural problems have often been explored using the cross‐lagged panel model (CLPM; Hipwell et al., 2008; Lansford et al., 2011; Serbin et al., 2015; Wang & Kenny, 2014). The CLPM estimates the effect of a predictor on an outcome while controlling for prior differences in the outcome. As such the CLPM can help to determine whether one variable *predicts* the other and/or vice versa. In these models, prospective longitudinal influences of one variable on another are referred to as cross‐lagged effects. However, CLPMs have several shortcomings when it comes to determining the direction of effects between associated variables (Hamaker et al., 2015). CLPMs cannot account for unmeasured sources of confounding, which may distort estimates of cross‐lagged effects and limits the capacity to draw causal inference from these models. Also, CLPMs do not distinguish within‐person changes from between‐person differences across repeated measures in the trait‐like, time‐invariant stability of many psychological constructs. As such, it is not possible to draw any conclusions on within‐person changes using CLPMs.

Two commonly used alternatives to the CLPM that are designed to strengthen the capacity for causal inference are the monozygotic twin difference cross‐lagged panel model (MZD‐CLPM; Moscati et al., 2018; Ritchie et al., 2015) and the random intercept‐cross‐lagged panel model (RI‐CLPM; Hamaker et al., 2015). The MZD‐CLPM and RI‐CLPM account for between‐subject differences across time and thus can control for potential sources of unobserved confounding. However, these models differ conceptually and in their underlying assumptions, so the conclusions that can be drawn from these methods also differ. Triangulating results across multiple (longitudinal) methods strengthens the ability to draw conclusions regarding causality. Converging evidence from these models would serve to reinforce confidence in any putative causal relationships between maladaptive parenting variables. In this study, we provide the reader with an overview of features of the MZD‐CLPM and RI‐CLPM (see Table 1) and examine associations between maladaptive parenting and child emotional and behavioural problems using both models in a dataset of MZ twins.

**TABLE 1:** Overview of model features of three CLPM models to test direction of effects in longitudinal data.

	CLPM	MZD‐CLPM	RI‐CLPM
Model type	Cross‐lagged panel model	MZ twin difference cross‐lagged panel model	Random intercept cross‐lagged panel model
Illustration of the model	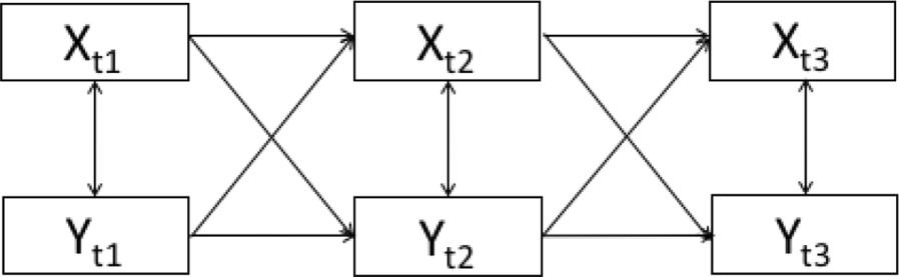	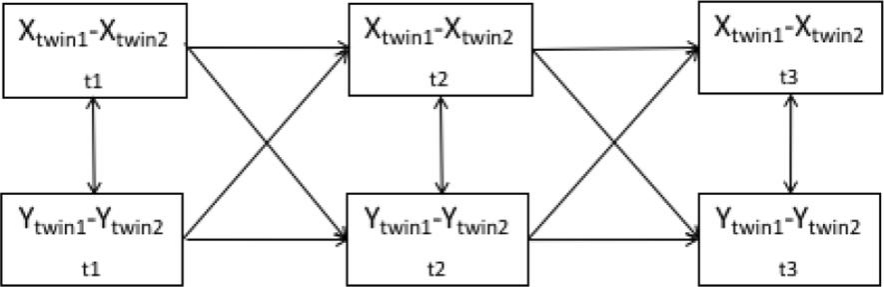	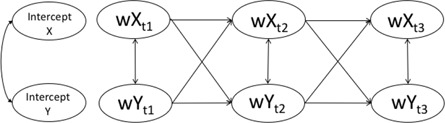
Model features	Cross‐lagged paths are intended to inform on direction of effects between variables over time.	Cross‐lagged paths are intended to inform on direction of effects between variables over time.	Cross‐lagged paths are intended to inform on direction of effects between variables over time.
	Cross‐lagged effects indicate to what extent for example, trait *X_t_* prospectively predicts trait *Y_t_*_+1_ controlling for autoregressive prediction from *Y_t_*, and vice versa.	Accounts for genetic and environmental effects shared by monozygotic twins by modelling relationships between twin differences in traits *X* and *Y*.	Accounts for stable between‐subject differences across development by inclusion of random intercepts.
		Cross‐lagged effects indicate to what extent within‐twin pair differences in *X_t_* prospectively predict the same in *Y_t_*_+1_, controlling for prior differences in *Y_t_* and vice versa.	Separation of between‐subject (time‐invariant) and within‐subject (time‐variant) variation.
		Differences between twins can be attributed to nonshared environmental influences unique to each twin.	Cross‐lagged effects indicate to what extent within‐person deviation from the trait level of *X_t_* prospectively predicts change in the within‐person deviation from the trait level of *Y_t_*_+1_, controlling for prior deviation at *Y_t_*_1_ and vice versa.
Controls for	Autoregressive effects and within‐time covariance.	Autoregressive effects and within‐time covariance.	Autoregressive effects and within‐time covariance.
		Any unobserved confounding effects indexed by the genetic and environmental influences shared by MZ twins. Confounding influences accounted for in this model will include stable and time‐variant effects.	Any unobserved confounding effects indexed by stability within a trait across time (i.e., trait‐like, *time‐invariant* stability). Confounding effects accounted for in this model will be time‐invariant.
Example RQ	Are children who report higher levels of maladaptive parenting (compared to others in the sample) at age 9 also likely to show more behavioural problems (compared to others in the sample) at age 12?	Are children who report higher levels of maladaptive parenting (compared to their co‐twin) at age 9 also likely to show more behavioural problems (compared to their co‐twin) at age 12?	Are children who report higher deviations from their own average of maladaptive parenting at age 9 also likely to show higher deviations from their own average of behavioural problems at age 12?
Limitations	Does not control for unobserved confounding.	Does not control for unobserved confounding that is not shared by twins. Such confounding may be stable and/or time varying.	Does not control for time‐varying sources of unobserved confounding within individuals.
	Does not distinguish between‐person effects from within‐person processes.		
	Cannot be used to investigate whether the change in one variable between two measurement occasions is associated with change in another.		
Example of conclusion that can be drawn on behavioural problems and maladaptive parenting	‘When an individual reports more behavioural problems at Tx (relative to others), (s)he will be more likely to experience a subsequent rank‐order increase in maladaptive parenting at Tx + 1’.	‘When an individual reports more behavioural problems than his/her MZ cotwin at Tx, (s)he will be more likely to experience more maladaptive parenting as compared to their cotwin at Tx + 1. Moreover, any association will be explained by environmental factors unique to that child’.^a^	‘When an individual reports more behavioural problems than expected at Tx based on that individual's average (intercept), (s)he will be more likely to experience more maladaptive parenting than average at Tx + 1’.

*Note*: *X* = observed phenotype ‘*X*’, *Y* = observed phenotype ‘*Y*’, *t*1 = first measurement occasion, *t*2 = second measurement occasion, *t*3 = third measurement occasion, *X*_twin1_ − *X*_twin2_ = difference scores of *X* between twins in a pair, *Y*_twin1_ − *Y*_twin2_ = difference scores of *Y* between twins in a pair, w*X* = within‐person deviation from the trait level of *X*, w*Y* = within‐person deviation from the trait level of *Y*.

^a^ Irrespective of their rank position in the total distribution.

### MZ twin difference method

Fifty years of twin studies have shown that the heritability across all human traits is 49% (Polderman et al., 2015) and that most correlations between phenotypes are at least in part accounted for by genetic overlap (Plomin et al., 2016). In recent years genomic studies have lent support to the importance of genetic factors in explaining the variance in, and covariance between, human traits (Cheesman et al., 2020; Hagenaars et al., 2016; Jami et al., 2022; Strawbridge et al., 2018). Accounting for potential genetic confounding between traits should therefore be considered key in any research aimed at drawing causal inferences. MZ twins offer an opportunity to do this. MZ twins who grow up together share 100% of their genes and many aspects of their rearing environment. As such, when twins differ on an exposure of interest, they can each act as an almost‐perfect matched control for the other (McAdams et al., 2021; Pingault et al., 2018). In the MZ twin difference method, difference scores between twins in a pair are calculated for each variable of interest (e.g., differences between twins' perceived maladaptive parenting; and emotional and behavioural problems). These difference scores can then be used as predictors of one another (e.g., we can test whether twin differences in exposure to maladaptive parenting predict twin differences in behavioural problems). Cross‐lagged relationships between twin differences index the association between variables after controlling for genetic and environmental influences shared by MZ twins. Twin differences in one variable prospectively predicting differences in another, indicates that the population‐level association between the two variables is not entirely attributable to genetic or environmental confounding shared by MZ twins. A limitation of the MZ twin difference method is that environmental influences not‐shared between twins (i.e., ‘unique’ or ‘child‐specific’ environmental effects) are not accounted for and can still serve to confound associations. Longitudinal studies of MZ twins have demonstrated that negative parenting experiences are associated with increased child behavioural problems over time, even after accounting for potential genetic and shared environmental confounds (Burt et al., 2006; Cecil et al., 2012; Larsson et al., 2008; Lynch et al., 2006; Oliver, 2015; Viding et al., 2009).

### The random intercept CLPM (RI‐CLPM)

The MZ twin difference method can only be used on MZ twin data, so questions may remain about the generalisability of results to non‐twin populations. Another approach to strengthening researchers' ability to draw causal inferences that can be applied to non‐twin data is the RI‐CLPM (see Table 1 for details). The RI‐CLPM is intended to account for time‐invariant unobserved confounding by accounting for stable between‐subject differences across development by inclusion of random intercepts (Rohrer & Murayama, 2023; Usami et al., 2019). The residual within‐person variance on each variable (which indicates variation around a person's mean) is used to evaluate whether the relative position of one variable at one time point (distance above/below the intercept) predicts the relative position of another variable at the next time point. Thus, the focus in an RI‐CLPM is on *within‐person* variability. Cross‐lagged relationships between the residual within‐person variances of each variable therefore indexes within‐person effects after controlling for sources of between‐person variance (Hamaker et al., 2015). Controlling for stable (time‐invariant) between‐person differences, therefore strengthens the ability to draw causal inference from significant cross‐lags in the RI‐CLPM. A growing number of studies have investigated within‐person associations between parenting and various developmental outcomes with the RI‐CLPM, controlling for the between‐person variance (Aunola et al., 2013; Janssen et al., 2021; Nelemans et al., 2020). However, when distinguishing between‐ and within‐person associations there was very little evidence of time‐lagged within‐person effects between parenting and child problems (Boele et al., 2020).

### Comparing the MZD‐CLPM and the RI‐CLPM

The MZD‐CLPM and the RI‐CLPM have been proposed as ways of improving the capacity of the CLPM to control for unobserved sources of confounding. It is well‐known that identifying, measuring and controlling for potential confounders helps to delineate cause and effect in data analysis, but that it is challenging to capture all confounding effects in this manner. As such, any method that allows researchers to control for *unobserved* confounding strengthens our ability to draw causal inferences. Besides being designed for different types of data (MZ twins vs. singleton offspring), the MZD‐CLPM and RI‐CLPM also differ in the types of unobserved confounding they control for (see Table 1). The RI‐CLPM controls for time‐invariant confounding but does not control for time‐variant confounding. The MZD‐CLPM controls for effects shared between twins and does not control for nonshared effects. Thus, if the effects shared between twins are invariant across time, the MZD‐CLPM and the RI‐CLPM control for the same sources of unobserved confounding. One might be tempted to think that this is true. For example, MZ twins share their DNA, and DNA is time invariant. However, the effects of DNA can be time variant. Genetic innovation is commonly reported within the genetic literature, with the same trait being under the influence of different genetic factors during different developmental periods (Hannigan et al., 2017). Thus, to the extent that effects shared by twins are instable across time (or to the extent that nonshared effects are stable), the MZD‐CLPM and the RI‐CLPM control for different sources of confounding. The MZD‐CLPM controls for all genetic and environmental effects shared by twins, be they time variant or invariant. The RI‐CLPM controls for all stable effects. This will include stable genetic and environmental effects (including stable environmental effects not shared by twins) and will exclude time‐varying genetic and environmental effects.

### Present study

Since the MZD‐CLPM and RI‐CLPM control for overlapping but non‐identical sources of unmeasured confounding, comparing results from these two approaches will be informative and may aid researchers in understanding how and why findings converge/diverge when using distinct methods to control for unobserved confounding. The goal of the present study is to illustrate similarities and differences in findings and the conclusions that can be drawn using each method. Moreover, this study will help identify which results are robust and could inform clinical practice. We examine associations between maladaptive parenting and child emotional and behavioural problems in identical twins aged 9, 12 and 16 by applying both models within the same dataset. To the best of our knowledge, this is the first study to compare results obtained using an RI‐CLPM to those derived from an MZD‐CLPM.

## METHODS

### Sample

We examined data from 5698 monozygotic (MZ) twin pairs from the Twins Early Development Study (TEDS), an ongoing longitudinal study of twin pairs born between 1994 and 1996 in England and Wales. Participants were identified through birth records and approached for recruitment to the study (involving 16,810 families). The first TEDS data collection was conducted when twins were around 18 months of age. The sample is reasonably representative of the England and Wales population in terms of ethnic and socio‐economic diversity, as well as sex and zygosity of twins (Rimfeld et al., 2019). Overall, 55% of the MZ twin sample is female, and 93% of parents identified their twins as ‘white’ (using a single item with response options ‘Asian’, ‘Black’, ‘Mixed’, ‘White’ and ‘Other’). Participant retention in the complete TEDS sample is significantly associated with female sex, monozygosity and identifying racially as ‘white’. More detailed information on TEDS can be found elsewhere (Rimfeld et al., 2019). Here we focus on child rated measures, administered when the twins were aged 9, 12 and 16.

### Measures

#### Child problems

Emotional and behavioural problems were measured via child self‐report using the strengths and difficulties questionaire (SDQ, Goodman, 1997). The SDQ is designed to assess psychological adjustment in youth aged 3–16 years. The emotional problems subscale included five statements such as ‘I have many fears, I am easily scared’ and ‘I am nervous in new situations, I easily lose confidence’. The behavioural problems scale included five items such as ‘I am often accused of lying and cheating’, and ‘I fight a lot, I can make other people do what I want’ over the past 3 months. Ratings were on a three‐point scale (with response options ‘not true’ = 0, ‘somewhat true’ = 1 and ‘certainly true’ = 2). Sum scores on child self‐reported emotional problems and behavioural problems of the twins at age 9, 12 and 16 years were used separately in the analyses. Cronbach alpha's were as follows: EP9 = 0.686, BP9 = 0.592, EP12 = 0.682, BP12 = 0.597, EP16 = 0.694, BP16 = 0.536.

#### Maladaptive parenting

Maladaptive parenting was assessed by four items derived from the parenting domain of a semi‐structured interview (see Deater‐Deckard et al., 1998). Children reported how often parents used various disciplinary strategies to deal with instances of child misbehaviour (i.e., ‘When I misbehave I am smacked or slapped’; ‘When I misbehave Mum/Dad makes a joke out of it’). All items were answered on a 3‐point scale, with the options: ‘Not true’ = 0; ‘Quite true’ = 1; and ‘Very true’ = 2. The two positive items, ‘When I misbehave Mum/Dad is firm and calm with me’ and ‘When I misbehave Mum/Dad explains why what I have done is wrong’ were reverse‐coded, to ensure that higher scores reflect maladaptive parenting. Sum scores on child‐reported maladaptive parenting at age 9, 12 and 16 were used for the analyses. Although Cronbach's alphas were low (9 = 0.420, 12 = 0.455, 16 = 0.369), this is likely because the scale was made of only four items (alpha strongly depends on the number of items in a scale). Spearman's Rho correlations across years suggest that test–retest reliability is sufficient for this measure (9–12 years: *r*_s_ = 0.32, 9–16 years: *r*_s_ = 0.15, and 12–16 years: *r*_s_ = 0.28; see Table 2). Also, face validity of the measure is adequate and appropriate for the aim of the study to compare findings across two statistical models.

**TABLE 2:** Means, SDs and phenotypic Spearman's Rho correlations at age 9, 12 and 16.

		*M*	*SD*	1	2	3	4	5	6	7	8	9
1	Maladaptive parenting at 9	3.20	1.63	1								
2	Emotional problems at 9	3.22	2.40	0.157**	1							
3	Behavioural problems at 9	2.21	1.85	0.327**	0.330**	1						
4	Maladaptive parenting at 12	3.13	1.49	0.317**	0.077**	0.201**	1					
5	Emotional problems at 12	2.18	2.07	0.108**	0.381**	0.148**	0.124**	1				
6	Behavioural problems at 12	1.87	1.65	0.248**	0.206**	0.395**	0.293**	0.319**	1			
7	Maladaptive parenting at 16	3.12	1.35	0.138**	0.050	0.116**	0.283**	0.029	0.116**	1		
8	Emotional problems at 16	2.74	2.27	0.063*	0.237**	0.037	0.029	0.378**	0.083**	0.105**	1	
9	Behavioural problems at 16	1.56	1.40	0.125*	0.069**	0.210**	0.143**	0.124**	0.329**	0.180**	0.198**	1

*Note*: **Correlation is significant at the 0.01 level (2‐tailed). *Correlation is significant at the 0.05 level (2‐tailed).

### Statistical analyses

**FIGURE 1:**
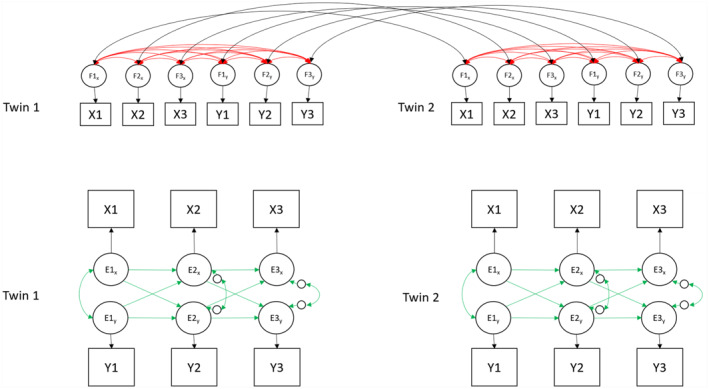
Path diagram of the MZD‐CLPM modelling variables ‘*X*’ and ‘*Y*’ across three timepoints. Top half of the figure depicts all familial influences shared between MZ twins and the bottom half depicts the MZ twin differences. Black double headed arrows are fixed to 1, reds are free, beta loadings are free, green arrows are equated across twins.

**FIGURE 2:**
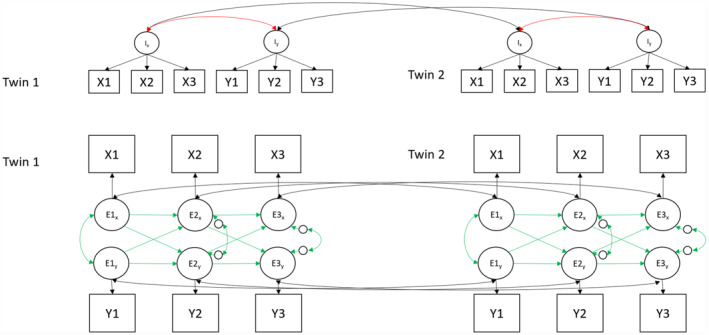
Path diagram of the RI‐CLPM modelling variables ‘*X*’ and ‘*Y*’ across three timepoints. Top half of the figure depicts between‐person effects and the bottom half depicts the within‐person effects. Black and red double headed arrows are freely estimated, beta loadings are free, green arrows are equated across twins.

Initially, a CLPM was specified to model the relationship between maladaptive parenting and child emotional and behavioural problems at 9, 12 and 16 years. Results from this model were intended to anchor our findings within the literature, which has predominantly used a CLPM to model bi/multivariate relationships over time (model 1). We then fitted a CLPM with MZ twin differences (model 2; MZD‐CLPM) and an RI‐CLPM (Hamaker et al., 2015) using the same data. In the MZD‐CLPM, we modelled latent familial factors to capture MZ twin covariance on traits. Residual variances captured twin differences. Autoregressive and cross‐lagged paths modelled covariance between these twin differences. A path diagram depicting our MZD‐CLPM can be found in Figure 1. In the RI‐CLPM, we accounted for non‐independence of twins by allowing twin 1 random intercepts to correlate with twin 2 random intercepts, and twin 1 within‐person residuals to correlate with twin 2 within‐person residuals. In this manner our MZD‐CLPM and our RI‐CLPM were each fitted to the same MZ twin dataset. This was done to aid comparison between the RI‐CLPM and the MZD‐CLPM. The path diagram of the RI‐CLPM is illustrated in Figure 2. Twin age and sex were regressed out of all variables and unstandardised residual scores were used in all models. Model fitting was carried out in R using the Lavaan‐package (Rosseel, 2012). We estimated all models using maximum likelihood estimation with robust standard errors for nonnormality of the data and ordinal scales of the measurements (MLR estimator) and full information maximum likelihood to deal with missing data. To evaluate model fit, we inspected χ^2^ test statistics, comparative fit index (CFI), standardised root mean square residuals (SRMR) and root mean square error of approximation (RMSEA). A CFI > 0.90 and a RMSEA < 0.08 are considered good (Hu & Bentler, 1999).

Our RI‐CLPM and MZD‐CLPM are not nested, so to inform the degree of correspondence between the RI‐CLPM and the MZD‐CLPM Spearman's Rho and R‐squared between beta coefficients were calculated. The analytic plan for this study was uploaded to Open Science Framework prior to analyses (https://osf.io/29s53/), we did not have access to the data prior to forming hypotheses and drawing the analytic plan.

#### Moderation analyses

Part of our pre‐registered analytic plan was to explore whether associations between maladaptive parenting and child emotional and behavioural problems differed between twins from families with high versus low levels of home chaos and socioeconomic status (SES). We ran two multiple group models, one to test home chaos and one to test SES as moderators. A more detailed description of the home chaos and SES constructs and the analytical models can be found in Appendix S3.

## RESULTS

### Descriptive statistics

Means, standard deviations and bivariate correlations among study variables are reported in Table 2. Spearman's Rho correlations indicated that maladaptive parenting, behavioural problems and emotional problems were significantly correlated with one another at all ages (range *r*_s_ = 0.12–0.33). Standard deviations and Spearman's Rho correlations of MZ difference scores are presented in Table 3. Within twin pair correlations of the study variables can be found in Table S1 (Appendix S1).

**TABLE 3:** SDs and Spearman's Rho correlations of MZ difference scores.

		*SD*	1	2	3	4	5	6	7	8	9
1	Twin difference score emotional problems at 9	2.52	1								
2	Twin difference score behavioural problems at 9	1.86	0.176**	1							
3	Twin difference score maladaptive parenting at 9	1.59	0.044*	0.131**	1						
4	Twin difference score emotional problems at 12	2.27	0.158**	0.071**	0.070**	1					
5	Twin difference score behavioural problems at 12	1.69	0.034	0.172**	0.014	0.221**	1				
6	Twin difference score maladaptive parenting at 12	1.52	0.000	0.069**	0.049*	0.063**	0.110**	1			
7	Twin difference score emotional problems at 16	2.33	0.102**	0.026	0.154**	0.206**	0.043*	0.029	1		
8	Twin difference score behavioural problems at 16	1.57	−0.018	0.071**	0.035	0.059**	0.154**	0.074**	0.140**	1	
9	Twin difference score maladaptive parenting at 16	1.45	0.027	0.048	0.015	0.016	0.026	0.071**	0.101**	0.067*	1

*Note*: **Correlation is significant at the 0.01 level (2‐tailed). *Correlation is significant at the 0.05 level (2‐tailed).

### Main analysis

We tested associations between maladaptive parenting, behavioural problems and emotional problems using the CLPM, MZD‐CLPM and RI‐CLPM. Informed by a comparison of model fit between constrained and unconstrained models, we specified that correlations and cross‐lagged effects were allowed to vary across time in all models. Model fit indices are presented in Table 4 and show all models fitted the data adequately. Estimated cross‐lagged paths and autoregressive effects of the CLPM can be found in Table S2 and Figure S1 (Appendix S2) as baseline information.

**TABLE 4:** Fit indices.

Model	Chi‐square	*df*	AIC	BIC	RMSEA	CFI	TLI
1. CLPM	40.0	9	92,385	92,675	0.027	0.989	0.957
2. MZD‐CLPM	114.0	99	90,060	90,578	0.008	0.997	0.995
3. RI‐CLPM	142.9	102	90,085	90,586	0.013	0.991	0.987

#### MZD‐CLPM

**FIGURE 3:**
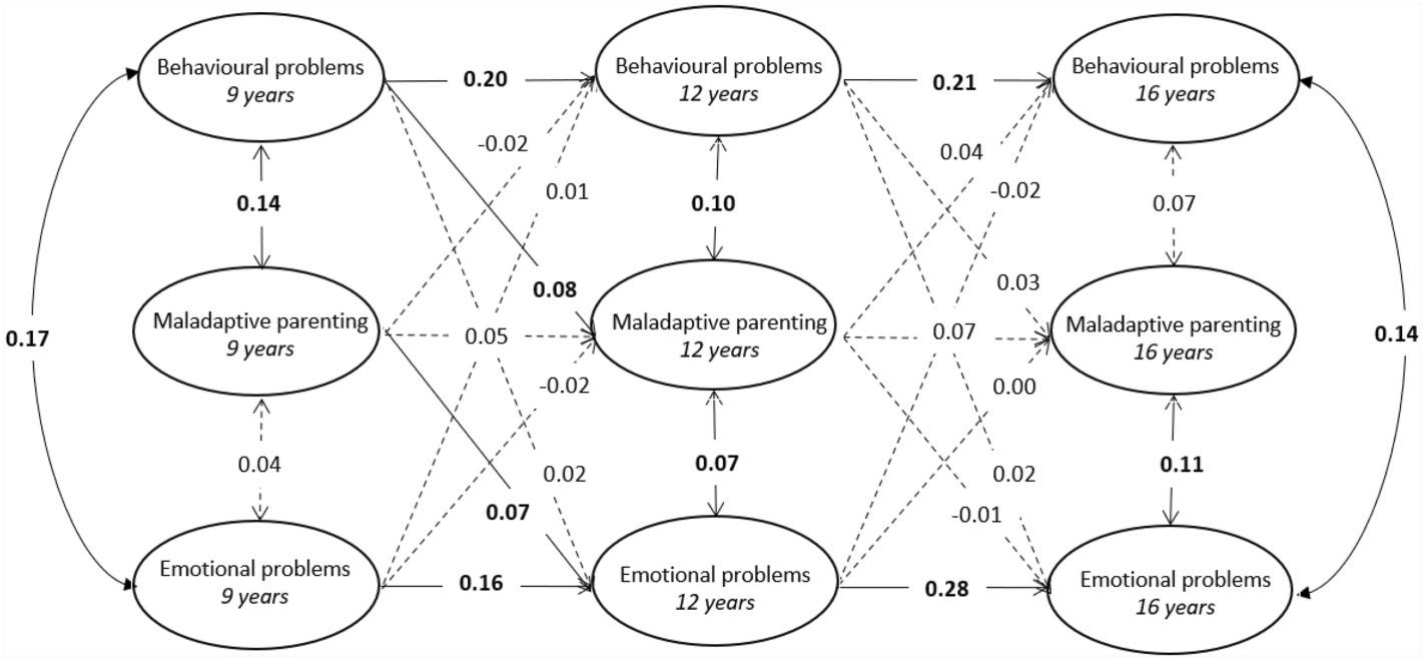
Representation of MZD‐CLPM with standardised effects (*β*). Covariance between behavioural problems and emotional problems at age 12 is missing in the depiction = 0.23. Estimates in **bold**/solid lines are significant (*p* < 0.05).

Results from the MZD‐CLPM is displayed in Figure 3 and Table 5 (estimates and 95% CI can be found in Table 5). Twin differences in behavioural problems at age 9 predicted twin differences in maladaptive parenting at age 12 (*β* = 0.08, *SE* = 0.03, *p* = 0.018) but not vice versa (*β* = −0.03 = 2, *SE* = 0.04, *p* = 0.488). We also found that twin differences in maladaptive parenting at age 9 predicted twin differences in emotional problems at age 12 (*β* = 0.10, *SE* = 0.05, *p* = 0.041), but not vice versa (*β* = −0.01, *SE* = 0.02, *p* = 0.544). All other cross‐lagged associations were non‐significant (*β* range [−0.01,0.04]).

**TABLE 5:** Parameter estimates.

Parameter	MZD‐CLPM				RI‐CLPM			
	Estimate	SE	*p*‐value	Std beta (95% CI)	Estimate	SE	*p*‐value	Std beta (95% CI)
Between‐person effects								
MP ↔ EP					0.30	0.10	**0.002**	0.50 (0.16, 0.85)
MP ↔ BP					0.28	0.07	**<0.001**	0.72 (0.44, 1.00)
EP ↔ BP					0.24	0.11	**0.030**	0.33 (0.09, 0.58)
Cross‐lagged effects								
MP9 → EP12	0.10	0.05	**0.041**	0.07 (0.00, 0.14)	−0.01	0.05	0.835	−0.01 (−0.10, 0.08)
MP9 → BP12	−0.03	0.04	0.488	−0.02 (−0.09, 0.04)	0.04	0.03	0.163	0.05 (−0.02, 0.11)
EP9 → MP12	−0.01	0.02	0.544	−0.02 (−0.08, 0.04)	−0.04	0.02	0.082	−0.06 (−0.13, 0.01)
EP9 → BP12	0.01	0.02	0.683	0.01 (−0.05, 0.08)	0.08	0.02	**0.001**	0.12 (0.05, 0.19)
BP9 → PD12	0.06	0.03	**0.018**	0.08 (0.01, 0.14)	0.09	0.03	**0.001**	0.11 (0.04, 0.18)
BP9 → EP12	0.02	0.04	0.587	0.02 (−0.05, 0.08)	0.13	0.04	**0.003**	0.13 (0.04, 0.21)
MP12 → EP16	−0.02	0.06	0.723	−0.01 (−0.09, 0.06)	−0.11	0.06	**0.049**	−0.08 (−0.16, 0.00)
MP12 → BP16	0.04	0.04	0.321	0.04 (−0.04, 0.11)	−0.01	0.04	0.797	−0.01 (−0.10, 0.08)
EP12 → MP16	0.00	0.03	0.939	0.00 (−0.08, 0.08)	−0.07	0.03	**0.033**	−0.10 (−0.18, −0.01)
EP12 → BP16	−0.01	0.03	0.698	−0.02 (−0.10, 0.07)	0.00	0.03	0.935	0.00 (−0.11, 0.10)
BP12 → MP16	0.03	0.04	0.438	0.03 (−0.05, 0.12)	0.03	0.04	0.534	0.03 (−0.06, 0.12)
BP12 → EP16	0.02	0.06	0.700	0.02 (−0.07, 0.10)	0.09	0.06	0.126	0.07 (−0.02, 0.17)
Contemporaneous associations								
EP9 ↔ BP9	0.40	0.08	**<0.001**	0.17 (0.10, 0.23)	1.121	0.14	**<0.001**	0.34 (0.27, 0.40)
EP9 ↔ MP9	0.08	0.06	0.215	0.04 (−0.02, 0.10)	0.34	0.13	**0.007**	0.11 (0.03, 0.18)
BP9 ↔ MP9	0.20	0.05	**<0.001**	0.14 (0.07, 0.20)	0.67	0.10	**<0.001**	0.26 (0.20, 0.32)
EP12 ↔ BP12	0.42	0.05	**<0.001**	0.23 (0.17, 0.28)	0.74	0.09	**<0.001**	0.31 (0.25, 0.38)
EP12 ↔ MP12	0.11	0.04	**0.011**	0.07 (0.02, 0.11)	0.09	0.08	0.277	0.04 (−0.03, 0.11)
BP12 ↔ MP12	0.12	0.03	**<0.001**	0.10 (0.05, 0.15)	0.32	0.06	**<0.001**	0.17 (0.12, 0.23)
EP 16 ↔ MP16	0.19	0.07	**0.005**	0.11 (0.04, 0.19)	0.13	0.09	0.130	0.06 (−0.02, 0.13)
EP16 ↔ BP16	0.23	0.07	**0.001**	0.14 (0.06, 0.21)	0.40	0.09	**<0.001**	0.20 (0.12, 0.28)
BP16 ↔ MP16	0.07	0.04	0.063	0.07 (0.00, 0.14)	0.13	0.06	**0.023**	0.10 (0.02, 0.18)
Autoregressions								
MP9 → MP12	0.05	0.03	0.143	0.05 (−0.02, 0.12)	0.17	0.04	**<0.001**	0.20 (0.12, 0.27)
MP12 → MP16	0.07	0.04	0.088	0.07 (−0.01, 0.15)	0.15	0.05	**0.002**	0.16 (0.60, 0.27)
EP9 → EP12	0.14	0.03	**<0.001**	0.16 (0.09, 0.23)	0.14	0.04	**0.001**	0.17(0.07, 0.28)
EP12 → EP16	0.29	0.05	**<0.001**	0.28 (0.19, 0.37)	0.20	0.07	**0.002**	0.19 (0.07, 0.31)
BP9 → BP12	0.18	0.03	**<0.001**	0.20 (0.13, 0.27)	0.20	0.04	**<0.001**	0.23 (0.15, 0.31)
BP12 → BP16	0.18	0.04	**<0.001**	0.21 (0.13, 0.28)	0.13	0.05	**0.005**	0.17 (0.06, 0.28)

*Note*: *p*‐values in bold are significant (*p* < 0.05).

Abbreviations: MP, maladaptive parenting; EP, emotional problems; BP, behavioural problems.

#### RI‐CLPM

**FIGURE 4:**
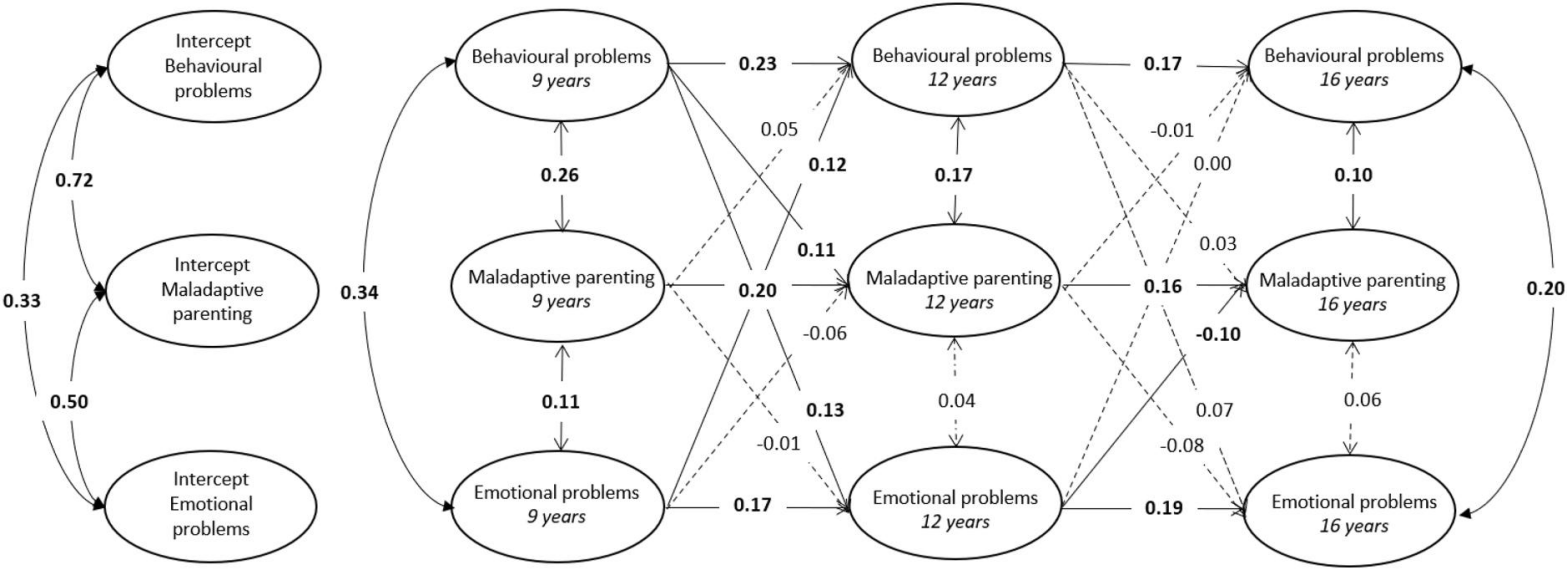
Simplified representation of the unconstrained RI‐CLPM with standardised effects (β). Covariance between behavioural problems and emotional problems at age 12 is missing in the depiction = 0.31. Estimates in **bold**/solid lines are significant (*p* < 0.05).

Results from the RI‐CLPM are displayed in Figure 4 and Table 5 (estimates and 95% CI can be found in Table 5). At the between‐person level, significant positive associations were found among maladaptive parenting, behavioural problems and emotional problems (MP‐EP; *β* = 0.50, *SE* = 0.10, *p* = 0.002, MP‐BP; *β* = 0.72, *SE* = 0.07, *p* = <0.001, EP‐BP; *β* = 0.33, *SE* = 0.11, *p* = 0.030). This indicates that children reporting higher levels of maladaptive parenting across all ages reported more behavioural problems and emotional problems as well. At the within‐person level, behavioural problems at age 9 were associated with maladaptive parenting at age 12 (*β* = 0.11, *SE* = 0.03, *p* = 0.001) but not vice versa (*β* = 0.05, *SE* = 0.03, *p* = 0.163). We did not find a significant within‐person association between maladaptive parenting at age 9 and emotional problems at age 12 (*β* = −0.01, *SE* = 0.05, *p* = 0.835) nor vice versa (*β* = −0.06, *SE* = 0.02, *p* = 0.082). From age 12 to 16, we found that emotional problems were predictive of decreased maladaptive parenting at age 16 (*β* = −0.10, *SE* = 0.03, *p* = 0.033). All other cross‐lagged associations between maladaptive parenting and child problems were non‐significant (*β* range [0.00, 0.07]).

Results from an MZD‐CLPM modelling associations between difference scores (calculated as twin 1 − twin 2) and from an RI‐CLPM clustering on family ID (an alternative way of accounting for non‐independence between twin) are included in Table S3 (Appendix S3) as these are the standard modelling approaches taken in the literature. We do not present them here because they cannot be compared in a direct manner (they are not fitted on exactly the same data). Results from these alternatively specified MZD‐CLPM and RI‐CLPM are nearly identical to those obtained from our main analyses presented above (all conclusions regarding the magnitude and significance of parameters were unchanged).

#### Comparing the MZD‐CLPM and RI‐CLPM

**FIGURE 5:**
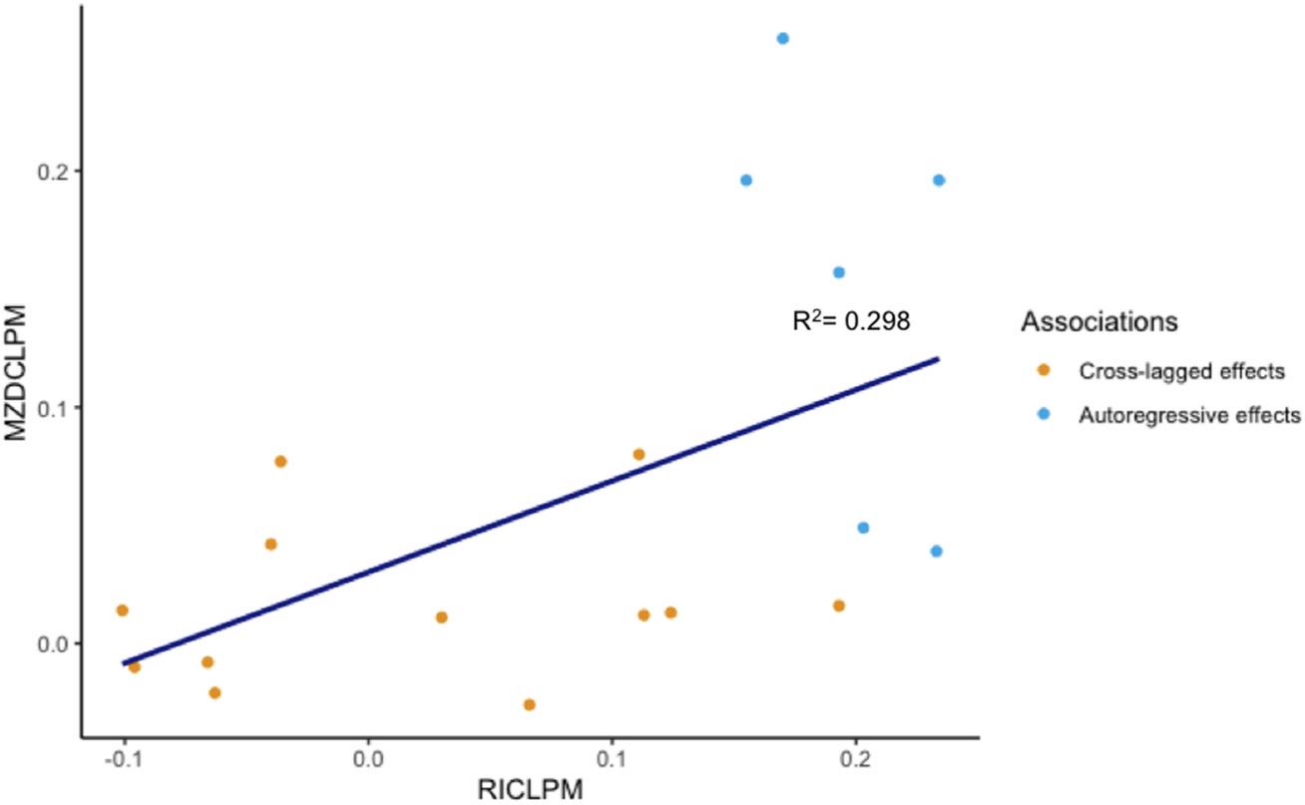
Beta coefficients of the regression effects from the MZD‐CLPM and RI‐CLPM.

The RI‐CLPM is not nested within the MZD‐CLPM. To estimate similarity between results of each model, we calculated correlations between the beta coefficients estimated in the MZD‐CLPM and RI‐CLPM. Spearman's Rho and R‐squared (*r*_s_ = 0.594, *p* = 0.009, *R* ^2^ = 0.298) indicated that the coefficients from each model were significantly correlated (see Figure 5). However, focussing only on the cross‐lagged estimates this was not the case, *r*_s_ = 0.273, *p* = 0.391, *R* ^2^ = 0.031.

### Preregistered post hoc analyses

We explored whether the associations between maladaptive parenting and child emotional problems and behavioural problems differed between twins from families with high versus low levels of home chaos and high versus low levels of SES. This was not the case in our study. Results from these analyses can be found in Appendix S4. Findings from our post hoc models, separating emotional and behavioural problems, are also presented in the supporting information (Appendix S5, Tables S4 and S5). Post hoc analysis suggested that overall the magnitude of associations was larger as compared to the findings from the multivariate models, but the pattern and direction of findings was similar.

## DISCUSSION

In this study we investigated longitudinal relationships between maladaptive parenting, and child emotional and behavioural problems, triangulating evidence across two variations of the CLPM: the MZD‐CLPM and the RI‐CLPM. Child emotional and behavioural problems are influenced by both stable and time‐varying genetic and environmental factors (Hannigan et al., 2017; Nivard et al., 2015), which can act as sources of unobserved confounding and cannot easily be accounted for in CLPMs. The MZD‐CLPM and RI‐CLPM control for (overlapping but non‐identical) sources of unobserved confounding and therefore enable us to draw stronger conclusions about potential causal processes operating between maladaptive parenting and child emotional and behavioural problems. One longitudinal cross‐lagged association was significant in both models: children's behavioural problems at age 9 predicted increased maladaptive parenting at age 12. The converging evidence from the MZD‐CLPM and RI‐CLPM strengthens our confidence in a potentially causal relationship between child behavioural problems at age 9 and consequent experienced maladaptive parenting at age 12. For all other cross‐lagged associations there was a lack of convergence across models. We discuss the meaning of convergence and divergence of the findings from the MZD‐CLPM and RI‐CLPM below.

### Why do findings from the MZD‐CLPM and RI‐CLPM not converge?

A comparison of beta coefficients from the RI‐CLPM and MZD‐CLPM (Figure 5) indicated a similar pattern of results from both models: beta coefficients were positively correlated and most associations were in the same direction. Nonetheless, substantial differences in the magnitude of associations estimated in each model were also evident. One noteworthy difference between models was that the MZD‐CLPM had far fewer significant paths than the RI‐CLPM. Differences between results derived from the RI‐CLPM and MZD‐CLPM likely exist because the two approaches control for non‐identical confounding influences. The MZD‐CLPM controls for all genetic factors and all environmental influences shared between MZ twins, these can be both time‐invariant (stable) and time‐varying (unstable). The random intercept of the RI‐CLPM, controls for time‐invariant (stable) effects, which could entail genetic and environmental influences shared between twins, but could also include stable non‐shared environmental influences. The RI‐CLPM does not account for time‐variant (unstable) within‐person confounding effects. Although genetic influences on traits are sometimes considered stable, evidence shows change over time, certainly across childhood and adolescence (Hannigan et al., 2017). As such, RI‐CLPM cross‐lagged estimates could include genetic effects shared between twins (see also Table 1 for an overview). Our results underline the subtle differences in the unobserved sources of confounding that these models account for, and how this can lead to important differences in the pattern of results and subsequent conclusions drawn. Clearly, there are important nuances to consider when drawing conclusions when using each of these distinct and complementary approaches (Lüdtke & Robitzsch, 2022, and see also Table 1).

Associations between traits that change over time, or traits that are only in part genetically linked (e.g., parenting experiences, child emotional and behavioural problems) may substantially vary across the MZD‐CLPM and RI‐CLPM. In the RI‐CLPM, we are unable to capture developmental changes of the child and their environment, as the model controls for time‐invariant confounding only. It could also be expected that the models might converge *more* when modelling associations among traits in adulthood, when we might expect genetic and environmental effects to be *more* stable. As a result, findings from the MZD‐CLPM and RI‐CLPM may be more similar when modelling traits that are stable over time and under stable genetic influence.

### What does it mean for causal hypotheses if findings from the MZD‐CLPM and RI‐CLPM diverge versus converge?

The fact that findings can diverge across methods ostensibly designed to do the same thing should be taken as a reminder that causal inference methods all have their own assumptions and limitations and cannot definitively test causal hypotheses on their own. However, where findings converge across methods, this should serve to reinforce confidence in any putative causal relationships between variables. For example, where an association is significant in both the MZD‐CLPM and the RI‐CLPM (as was the case for the path running from behavioural problems at age 9 to maladaptive parenting at age 12) the association can be said to remain after controlling for time‐invariant between‐person sources of unobserved confounding *and* after controlling for familial sources of unobserved confounding shared by MZ twins. It seems reasonable to interpret this as evidence supportive of a causal effect of behavioural problems on maladaptive parenting in childhood. Similarly, where paths are non‐significant in both the MZD‐CLPM and the RI‐CLPM, it seems reasonable to interpret this as evidence that causal effects are unlikely to be present, or are too small to be detected with our sample.

Divergence between models is more difficult to interpret than convergence but suggests we should be cautious in arriving at firm conclusions about whether we should or not draw causal inferences. In our example, the MZD‐CLPM seemed to be the more conservative approach, so several paths significant in the RI‐CLPM were not significant in the MZD‐CLPM. These paths (that were significant in the RI‐CLPM but not in the MZD‐CLPM) capture time‐variant influences shared between twins: genetic or shared environmental effects that influenced change. An important question is: should such paths be interpreted as supporting the notion that one variable causally influences another? One could argue that where one variable causally influences another, non‐shared environmental effects should be detectable on the path between those variables (as well as genetic or shared environmental effects). This is because all variables are under the influence of non‐shared environmental effects (Turkheimer, 2000), so any causal paths between variables should typically include non‐shared effects. For example, if *X* causes *Y*, and *X* is influenced by non‐shared effects, then the causal path between *X* and *Y* should capture some of the nonshared effects on *X*. Such non‐shared environmental effects would be picked up in the MZD‐CLPM (whether they were stable or time‐varying). It could therefore be that paths that are significant in the RI‐CLPM but not the MZD‐CLPM are attributable to time‐varying sources of confounding shared by twins: genetic or shared environmental effects that influence two variables and make them correlated. One example of a path that was significant in the MZD‐CLPM but not in the RI‐CLPM is the path running from maladaptive parenting at age 9 to emotional problems at age 12. In this case the effect is attributable to time‐invariant effects unshared between twins. This stable nonshared environmental effect could index a source of confounding not shared by twins.

### Limitations and future studies

Some limitations to our study should be noted. First, we would like to underline that we did find evidence for contemporaneous associations between maladaptive parenting and child emotional and behavioural problems. So, we do not know whether non‐significant findings regarding the time‐lagged effects indicate an absence of longitudinal associations or whether, for instance, the effects of maladaptive parenting and child problems unfold on a shorter time scale than our data permitted us to examine (Bolger & Laurenceau, 2013). Related to this, some researchers suggest that the RI‐CLPM may be more appropriate to use when answering questions about short‐term within‐person effects using time series data and/or intensive longitudinal designs. The RI‐CLPM may be less suited for understanding long‐term changes in longitudinal data with fewer measurements and long intervals as within‐person effects in the RI‐CLPM are based on scores that capture temporary fluctuations around a person's mean (Lüdtke & Robitzsch, 2022; Orth et al., 2021). The counterargument to this notion is that decomposition of the observations into a stable between‐person component and temporal within‐person components with the RI‐CLPM is independent of the time scale at which measurements were obtained and so can be used to study fluctuations over the short‐ and/or long‐term (Mulder, 2022). The significant cross‐lags from the RI‐CLPM in our study and in other studies with long intervals (Masselink et al., 2018; Nelemans et al., 2020; Schulz et al., 2021) provides support that predictions across long intervals can be observed. Second, factorial invariance of the measurements in our RI‐CLPM was not met (see Appendix S6, Table S6). This means that factor loadings are noninvariant over time which limits our ability to compare the latent variables and the associations across timepoints (Chen et al., 2005). Besides, changes in constructs during child development, certainly over a long‐time interval as we assess, is the reality (see also Hannigan et al., 2017). Thus, it could be expected that, in the RI‐CLPM, cross‐lagged associations between age 9 and 12 do not have the exact same meaning as cross‐lagged associations between age 12 and 16. With regard to our main study aim, comparison of the MZD‐ and RI‐CLPM, it can assumed that our corresponding conclusions are not affected by the fact that factorial invariance is not met.

Third, to compare the MZD‐CLPM cross‐lagged path estimates with those of the RI‐CLPM we assessed the correlation among 12 beta estimates. Low statistical power due to the low number of data points will have contributed to the non‐significance of some of these correlations. Our comparison of the models in this manner has an illustrative purpose and should be interpreted with caution. Fourth, using child self‐reports of problems and parenting raises the possibility that observed effects are inflated by shared method variance. The associations between child problems and maladaptive parenting should therefore be interpreted with this limitation in mind. In line with previous research (De Los Reyes & Kazdin, 2005), parent and child reports of the child problems in our study show low‐to‐moderate levels of agreement: EP age 9 = 0.40 (*p* < 0.001), BP age 9 = 0.41 (*p* < 0.001), EP age 12 = 0.44 (*p* < 0.001), BP age 12 = 0.46 (*p* < 0.001), BP age 16 = 0.43 (*p* < 0.001). Parent reports were not available for all measures. Therefore, we could not triangulate our entire models by using parent reports. We encourage future studies to include child and parent reports to control for potential reporter effects. Fifth, our measure of maladaptive parenting scored low on Cronbach's alpha, indicating low reliability. When controlling for confounding by using MZ twin differences, all error (which, if random, is not shared between twins) is included in the twin differences. This reduces the magnitude of the correlation between twin difference scores, because we are not estimating the correlation between the non‐shared environmental influence (*E*) on variable *X* and the *E* of variable *Y*, but rather *E*+ error of each. Assuming random measurement error, this means that the residual within‐time or within‐twin pair effects might be attenuated. However, face validity of the parenting measure is reasonable, test–retest reliability and inter‐item correlations (Appendix S7, Figure S2 and Table S7) seemed sufficient and the measure correlated with emotional and behavioural problems as expected. It can be assumed that the maladaptive parenting items measured a stable trait‐like phenotype and it is therefore expected that the measure is appropriate for the aim of the current study to compare models and that our findings are robust and reliable. Findings should be replicated (using measures of maladaptive parenting including more items) to further strengthen confidence in the associations between child problems and maladaptive parenting. Lastly, in future studies it would be interesting to account for developmental changes using a random slope, which can be driven by genetic, shared environmental or non‐shared environmental influences, by adding a random slope to the RI‐CLPM. We were unable to do so in the present study without at least four waves of data (Mund & Nestler, 2019).

## CONCLUSIONS

Taken together, our study described the types of unmeasured confounding different longitudinal designs can account for, and underlined how slight differences in the sort of confounding being controlled for can lead to quite different conclusions, even when using the exact same data (see Table 1). Findings from all models, that is, the CLPM, RI‐CLPM and MZD‐CLPM, indicated that child behavioural problems at age 9 predicted increased maladaptive parenting at age 12. These results can be interpreted as corroborating (albeit not conclusive) evidence in favour of a causal relationship.

Importantly however, results also illustrate divergence in the MZD‐CLPM and RI‐CLPM outcomes. While both methods are intended to improve the ability of researchers to draw causal inference, they do not lead to the same conclusions. The substantial differences in results underline that nuance is required when interpreting findings using such models and that triangulating results across multiple (longitudinal) methods strengthens the ability to draw conclusions regarding causality.

## AUTHOR CONTRIBUTIONS

Marie‐Louise J. Kullberg: Formal analysis; investigation; writing – original draft. Charlotte C. Van Schie: Supervision; writing – review and editing. Andrea G. Allegrini: Methodology; writing – review and editing. Yasmin Ahmadzadeh: Writing – review and editing. Daniel L. Wechsler: Writing – review and editing. Bernet M. Elzinga: Supervision; writing – review and editing. Tom A. McAdams: Conceptualisation; methodology; supervision; writing – review and editing.

## CONFLICT OF INTEREST STATEMENT

Tom A. McAdams is on the JCPP Advances Editorial Advisory Board. The remaining authors have declared that they have no competing or potential conflicts of interest.

## ETHICS CONSIDERATIONS

No ethical approval was required for this article.

## Supporting information

Supporting Information S1

## Data Availability

The data used and described in this paper came from the Twins Early Development Study (TEDS). Researchers can request access to TEDS data: https://www.teds.ac.uk/researchers/teds‐data‐access‐policy.
