# Supplementary material for: Comparing findings from the random‐intercept cross‐lagged panel model and the monozygotic twin difference cross‐lagged panel model: Maladaptive parenting and offspring emotional and behavioural problems
Source: JCPP Adv. 2023 Oct 28;4(1):e12203. doi: 10.1002/jcv2.12203 (PMC10933702; doi:10.1002/jcv2.12203)
Supplement: Supplementary file 1 — Supporting Information S1 [file JCV2-4-e12203-s001.docx]

**Supplementary Materials**

**Appendix 1. Within MZ twin pair correlations**

Table S1. Within MZ twin pair correlations

|  |  | 1 | 2 | 3 | 4 | 5 | 6 | 7 | 8 | 9 |  |  |  |  |  |  |
| --- | --- | --- | --- | --- | --- | --- | --- | --- | --- | --- | --- | --- | --- | --- | --- | --- |
| 1 | Behavioural problems age 9 | .483** |  |  |  |  |  |  |  |  |  |  |  |  |  |  |
| 2 | Emotional problems age 9 | .319** | .446** |  |  |  |  |  |  |  |  |  |  |  |  |  |
| 3 | Maladaptive parenting age 9 | .329** | .139** | .528** |  |  |  |  |  |  |  |  |  |  |  |  |
| 4 | Emotional problems age 12 | .146** | .398** | .119** | .394** |  |  |  |  |  |  |  |  |  |  |  |
| 5 | Behavioural problems age 12 | .401** | .196** | .221** | .329** | .474** |  |  |  |  |  |  |  |  |  |  |
| 6 | Parental discipline age 12 | .225** | .101** | .299** | .135** | .243** | .478** |  |  |  |  |  |  |  |  |  |
| 7 | Emotional problems age 16 | .046 | .202** | .000 | .376** | .042* | .003 | .471** |  |  |  |  |  |  |  |  |
| 8 | Behavioural problems age 16 | .202** | .085** | .075** | .126** | .230** | .090** | .136** | .372** |  |  |  |  |  |  |  |
| 9 | Parental discipline age 16 | .123** | .026 | .136** | .040 | .094** | .250** | .063* | .142** | .435** |  |  |  |  |  |  |
| ** Correlation is significant at the 0.01 level (2-tailed), * Correlation is significant at the 0.05 level (2-tailed). | | | | | | | | | | | |  |  |  |  |  |

**Appendix 2. Findings from the CLPM**

Maladaptive parenting at age 9 prospectively predicted behavioural problems at age 12 and vice versa (*β*=0.10, *SE*=0.02, *p*<.001); behavioural problems at age 9 prospectively predicted maladaptive parenting at age 12 (*β*=0.14, *SE*=0.02, *p*<.001). Also at the next occasions, age 12 and 16, maladaptive parenting (*β*=0.06, *SE*=0.02, *p*=0.045) and behavioural problems (*β*=0.08, *SE*=0.08, *p*=0.019) predicted each other prospectively. No cross-lagged associations between emotional problems and maladaptive parenting were found (*β* range -0.02 - 0.04). Autoregressions and contemporaneous correlations were all significant in this model (*β* range 0.09 - 0.39). See Figure S1 and table S1 for model parameters.

*Figure S1.* Representation of CLPM with standardized effects (β). *Note.* Covariance between behavioural problems and emotional problems at age 12 = 0.27. Estimates in **bold** /solid lines are significant (*p*<.05).


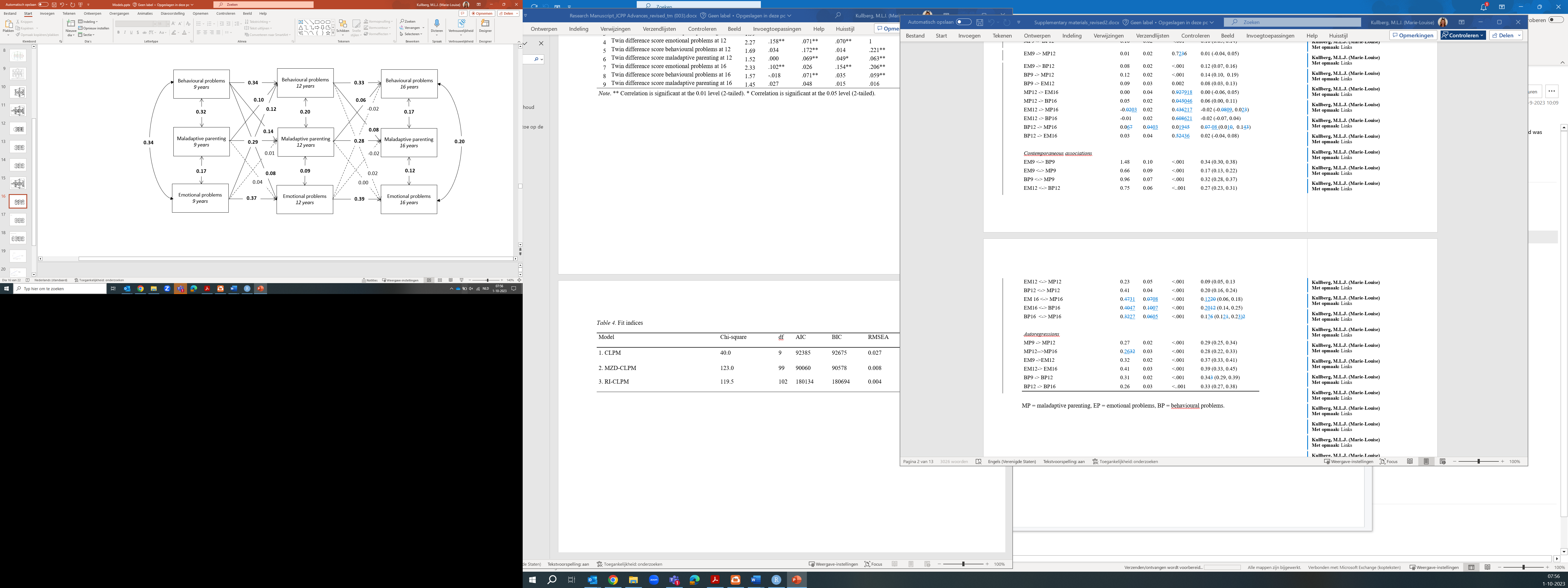


| Table S2. Parameter Estimates CLPM |  |  |  |  |
| --- | --- | --- | --- | --- |
| Parameter | Estimate | SE | p-value | Std beta (95% CI) |
| *Cross-lagged effects* |  |  |  |  |
| MP9 -> EM12 | 0.05 | 0.03 | 0.096 | 0.04 (-0.01, 0.09) |
| MP9 -> BP12 | 0.10 | 0.02 | <.001 | 0.10 (0.05, 0.14) |
| EM9 -> MP12 | 0.01 | 0.02 | 0.726 | 0.01 (-0.04, 0.05) |
| EM9 -> BP12 | 0.08 | 0.02 | <.001 | 0.12 (0.07, 0.16) |
| BP9 -> MP12 | 0.12 | 0.02 | <.001 | 0.14 (0.10, 0.19) |
| BP9 -> EM12 | 0.09 | 0.03 | 0.002 | 0.08 (0.03, 0.13) |
| MP12 -> EM16 | 0.00 | 0.04 | 0.918 | 0.00 (-0.06, 0.05) |
| MP12 -> BP16 | 0.05 | 0.02 | 0.046 | 0.06 (0.00, 0.11) |
| EM12 -> MP16 | -0.03 | 0.02 | 0.217 | -0.02 (-0.09, 0.02) |
| EM12 -> BP16 | -0.01 | 0.02 | 0.621 | -0.02 (-0.07, 0.04) |
| BP12 -> MP16 | 0.06 | 0.03 | 0.019 | 0.08 (0.01, 0.14) |
| BP12 -> EM16 | 0.03 | 0.04 | 0.36 | 0.02 (-0.04, 0.08) |
|  |  |  |  |  |
| *Contemporaneous associations* |  |  |  |  |
| EM9 <-> BP9 | 1.48 | 0.10 | <.001 | 0.34 (0.30, 0.38) |
| EM9 <-> MP9 | 0.66 | 0.09 | <.001 | 0.17 (0.13, 0.22) |
| BP9 <-> MP9 | 0.96 | 0.07 | <.001 | 0.32 (0.28, 0.37) |
| EM12 <-> BP12 | 0.75 | 0.06 | <..001 | 0.27 (0.23, 0.31) |
| EM12 <-> MP12 | 0.23 | 0.05 | <.001 | 0.09 (0.05, 0.13 |
| BP12 <-> MP12 | 0.41 | 0.04 | <.001 | 0.20 (0.16, 0.24) |
| EM 16 <-> MP16 | 0.31 | 0.08 | <.001 | 0.12 (0.06, 0.18) |
| EM16 <-> BP16 | 0.47 | 0.07 | <.001 | 0.20 (0.14, 0.25) |
| BP16 <-> MP16 | 0.27 | 0.05 | <.001 | 0.17 (0.12, 0.23) |
|  |  |  |  |  |
| *Autoregressions* |  |  |  |  |
| MP9 -> MP12 | 0.27 | 0.02 | <.001 | 0.29 (0.25, 0.34) |
| MP12-->MP16 | 0.26 | 0.03 | <.001 | 0.28 (0.22, 0.33) |
| EM9 ->EM12 | 0.32 | 0.02 | <.001 | 0.37 (0.33, 0.41) |
| EM12-> EM16 | 0.41 | 0.03 | <.001 | 0.39 (0.33, 0.45) |
| BP9 -> BP12 | 0.31 | 0.02 | <.001 | 0.34 (0.29, 0.39) |
| BP12 -> BP16 | 0.26 | 0.03 | <..001 | 0.33 (0.27, 0.38) |

MP = maladaptive parenting, EP = emotional problems, BP = behavioural problems.

**Appendix 3. MZD-CLPM modelling associations between difference scores (and from an RI-CLPM clustering on family ID**

| Table S3. Parameter Estimates MZD-CLPM using twin difference scores and RI-CLPM clustering on FamilyID | | | | | | | | | |  |
| --- | --- | --- | --- | --- | --- | --- | --- | --- | --- | --- |
|  | MZD-CLPM | | | | RI-CLPM | | | | |  |
| Parameter | Estimate | SE | p-value | Std beta (95% C) | | Estimate | SE | p-value | Std beta (95% CI) |  |
| *Between-person effects* |  |  |  |  | |  |  |  |  |  |
| MP <-> EM |  |  |  |  | | 0.33 | 0.11 | 0.003 | 0.65 (0.14, 1.17) |  |
| MP <-> BP |  |  |  |  | | 0.26 | 0.08 | 0.001 | 0.78 (0.40, 1.17) |  |
| EM <-> BP |  |  |  |  | | 0.31 | 0.12 | 0.007 | 0.41 (0.17, 0.65) |  |
|  |  |  |  |  | |  |  |  |  |  |
| *Cross-lagged effects* |  |  |  |  | |  |  |  |  |  |
| MP9 -> EM12 | 0.11 | 0.05 | 0.024 | 0.08 (0.01, 0.14) | | -0.02 | 0.05 | 0.705 | -0.02 (-0.10, 0.07) |  |
| MP9 -> BP12 | -0.03 | 0.04 | 0.436 | -0.03 (-0.09, 0.04) | | 0.07 | 0.03 | 0.030 | 0.11 (0.04, 0.19) |  |
| EM9 -> MP12 | -0.01 | 0.02 | 0.522 | -0.02 (-0.09, 0.04) | | -0.03 | 0.02 | 0.230 | -0.04 (-0.12, 0.03) |  |
| EM9 -> BP12 | 0.01 | 0.02 | 0.692 | 0.01 (-0.05, 0.08) | | 0.09 | 0.03 | 0.001 | 0.12 (0.05, 0.20) |  |
| BP9 -> MP12 | 0.07 | 0.03 | 0.015 | 0.08 (0.02, 0.14) | | 0.10 | 0.03 | 0.002 | 0.07 (0.01, 0.14) |  |
| BP9 -> EM12 | 0.01 | 0.04 | 0.716 | 0.01 (-0.05, 0.08) | | 0.12 | 0.05 | 0.009 | 0.11 (0.03, 0.20) |  |
| MP12 -> EM16 | -0.01 | 0.06 | 0.817 | -0.01 (0.09, 0.07) | | -0.10 | 0.06 | 0.124 | -0.07 (-0.16, 0.02) |  |
| MP12 -> BP16 | 0.04 | 0.04 | 0.254 | 0.04 (-0.03, 0.11) | | -0.01 | 0.04 | 0.761 | -0.02 (-0.13, 0.09) |  |
| EM12 -> MP16 | 0.00 | 0.03 | 0.923 | 0.00 (-0.08, 0.09) | | -0.09 | 0.04 | 0.012 | -0.12 (-0.21, -0.03) |  |
| EM12 -> BP16 | -0.01 | 0.03 | 0.861 | -0.01 (-0.09, 0.07) | | -0.04 | 0.04 | 0.315 | -0.06 (-0.18, 0.06) |  |
| BP12 -> MP16 | 0.02 | 0.04 | 0.688 | 0.02 (-0.07, 0.11) | | 0.03 | 0.04 | 0.536 | 0.03 (-0.06, 0.12) |  |
| BP12 -> EM16 | 0.02 | 0.06 | 0.700 | 0.02 (-0.07, 0.10) | | 0.06 | 0.06 | 0.362 | 0.04 (-0.05, 0.14) |  |
|  |  |  |  |  |  |  |  |  |  |  |
| *Contemporaneous associations* |  |  |  |  | |  |  |  |  |  |
| EM9 <-> BP9 | 0.80 | 0.16 | <.001 | 0.17 (0.10, 0.24) | | 1.17 | 0.13 | <.001 | 0.32 (0.26, 0.39) |  |
| EM9 <-> MP9 | 0.16 | 0.13 | 0.207 | 0.04 (-0.02, 0.10) | | 0.33 | 0.13 | 0.011 | 0.01 (0.02, 0.18) |  |
| BP9 <-> MP9 | 0.40 | 0.10 | <.001 | 0.13 (0.07, 0.20) | | 0.70 | 0.10 | <.001 | 0.27 (0.20, 0.33) |  |
| EM12 <-> BP12 | 0.83 | 0.10 | <.001 | 0.23 (0.17, 0.28) | | 0.70 | 0.09 | <.001 | 0.29 (0.23, 0.36) |  |
| EM12 <-> MP12 | 0.22 | 0.09 | 0.010 | 0.07 (0.02, 0.12) | | 0.08 | 0.09 | 0.378 | 0.03 (-0.04, 0.11) |  |
| BP12 <-> MP12 | 0.25 | 0.06 | <.001 | 0.10 (0.05, 0.15) | | 0.33 | 0.06 | <.001 | 0.18 (0.12, 0.23) |  |
| EM 16 <-> MP16 | 0.36 | 0.14 | 0.011 | 0.12 (0.03, 0.20) | | 0.11 | 0.10 | 0.281 | 0.05 (-0.04, 0.13) |  |
| EM16 <-> BP16 | 0.46 | 0.14 | 0.001 | 0.14 (0.06, 0.21) | | 0.28 | 0.29 | 0.003 | 0.15 (0.06, 0.24) |  |
| BP16 <-> MP16 | 0.13 | 0.08 | 0.108 | 0.17 (-0.02, 0.13) | | 0.11 | 0.07 | 0.090 | 0.09 (-0.01, 0.18) |  |
|  |  |  |  |  | |  |  |  |  |  |
| *Autoregressions* |  |  |  |  | |  |  |  |  |  |
| MP9 -> MP12 | 0.04 | 0.03 | 0.267 | 0.04 (-0.03, 0.11) | | 0.23 | 0.04 | <.001 | 0.25 (0.17, 0.33) |  |
| MP12-->MP16 | 0.07 | 0.04 | 0.088 | 0.07 (-0.01, 0.15) | | 0.19 | 0.05 | <.001 | 0.21 (0.10, 0.32) |  |
| EM9 ->EM12 | 0.14 | 0.03 | <.001 | 0.16 (0.09, 0.23) | | 0.16 | 0.05 | <.001 | 0.19 (0.09, 0.30) |  |
| EM12-> EM16 | 0.27 | 0.05 | <.001 | 0.26 (0.17, 0.34) | | 0.18 | 0.07 | 0.009 | 0.17 (0.04, 0.31) |  |
| BP9 -> BP12 | 0.18 | 0.03 | <.001 | 0.20 (0.12, 0.27) | | 0.21 | 0.04 | <.001 | 0.24 (0.15, 0.32) |  |
| BP12 -> BP16 | 0.18 | 0.04 | <.001 | 0.20 (0.12, 0.28) | | 0.11 | 0.05 | 0.017 | 0.16 (0.03, 0.28) |  |

**Appendix 4. Effects of socioeconomic status and home chaos**

**Background**

Socioeconomic disadvantages are associated with children’s emotional and behavioural problems (Bøe et al. ,2014; Flouri & Midouhas, 2017; Goodnight et al., 2012). Families’ socioeconomic status (SES), generally estimated using parents’ occupation and educational qualifications, reflects a family’s standing in the stratification system and determines their access to social and material resources (Winkleby et al., 1992). Previous research shows that children from families characterized by low SES displayed more behavioural problems as compared to children from high SES families (Hendriks et al. ,2020; Letourneau et al., 2013). Socioeconomic disadvantages are closely linked to measures of ‘chaos’ in the home environment (Dumas et al., 2005). Chaos in the home environment, or ‘home chaos’, reflects the level of disorganization within the household, including noise and lack of daily routines. According to a recent systematic review, there is consistent evidence that home chaos is associated with negative parenting practices and child’s social and behavioural problems (Marsh et al., 2020). Low SES might be associated with higher home chaos, negative parenting practices and child problems as lower social and financial support and higher life stress can exacerbate family life (McNeilly et al., 2021). However, the moderating effects of home chaos and SES remain unequivocal. Whereas one study confirmed that home chaos moderates the relation between hostile parenting and adolescent’s well-being two-years later (Tucker et al., 2018), others showed that reciprocal associations between harsh discipline and child’s externalising behaviour were not moderated by SES or home chaos (Lansford et al., 2011; Mills-Koonce et al., 2016). To examine whether the associations between maladaptive parenting and adolescents’ outcomes differ across families’ with low versus high SES and low versus high levels of home chaos, we added these two variables to our analyses as moderators.

**Method**

*Analyses*

*Socioeconomic status (SES)*

Families’ SES was based on mothers' and fathers' highest educational qualifications, their employment levels and mother’s age on birth of the first child. SES was assessed at first contact when the twins were 1.5 years old and at age 16. SES was used as a moderator in the analyses.

*Home Chaos*

Six items from the Confusion, Hubbub and Order Scale (CHAOS; Matheny et al., 1995) were used to assess the chaos at home. Items for parents included ‘the twins have regular bedtime routine’ and ‘we are usually able to stay on top of things’. Twin assessment consisted of items such as ‘You can’t hear yourself think in our home’. All items were rated on a 3-point scale (‘Certainly true’, ‘Somewhat true’ or ‘Not true’). Twin reports on chaos at home, assessed at the age of 9 years, were used as moderators in the analyses.

*Analyses*

SES at fist contact (model 4-6) and home chaos at age 9 (model 7-9) will be included as predictors of the random intercept to examine whether these factors explain differences between persons (Mulder & Hamaker, 2021). To test whether associations differ across (a) SES and (b) levels of home chaos, we ran multigroup models. Participants were equally divided into three groups (“low”, “moderate” and “high”) to compare whether associations between variables of interest differed across SES and levels of home chaos.

**Results**

We explored whether the associations between maladaptive parenting and adolecent emotional and behavioural problems differed between twins from families with high versus low levels of home chaos and high versus low levels of SES (aim 3).

First, we extended the RI-CLPM with SES as predictor of the random intercepts of maladaptive parenting and adolecent emotional and behavioural problems. Hihger SES was significantly associated with less maladaptive parenting (β=-0.23, *p*<.001), less emotional problems (β=-0.18, *p*<.001) and less behavioural problems (β=-0.31, *p*<.001). Next, we ran a multiple group model with SES a categorical variable, divided in low, moderate and high, to test whether the associations between maladaptive parenting, emotional and behavioural problems differed between high versus low SES families. The chi-square difference test indicated that the constrained model, fixing regressions to be equal across groups, did not differ from the unconstrained model (Δχ^2^=47.96, *p*=0.071), see *table 2* for fit indices. This suggests that the associations between maladaptive parenting and child problems did not differ across SES.

*Home chaos as a predictor of between-family differences*

To test whether the associations between maladaptive parenting and adolecent emotional and behavioural problems differ between twins from families with high versus low levels of home chaos (aim 3b), we ran similar models as for SES. Results from the extended RI-CLPM, including home chaos at age 9 as time-invariant predictor of the random intercepts, showed that higher levels of chaos at the home environment were associated with more maladaptive parenting (β=0.68, *p*<.001), more child emotional problems (β=0.43, *p*<.001) and more behavioural problems (β=0.72, *p*<.001). Results of the multiple group models with the categorized home chaos score (high, moderate, low) showed that the constrained model did not differ from the unconstrained model (Δ*χ*^2^=29.06, *p*=0.787), in *table 2* fit indices can be found. This means that the associations between maladaptive parenting and child problems were similar for adolescents from households with low, moderate and high levels of home chaos

**References**

Bøe, T., Sivertsen, B., Heiervang, E., Goodman, R., Lundervold, A. J., & Hysing, M. (2014). Socioeconomic status and child mental health: The role of parental emotional well-being and parenting practices. *Journal of abnormal child psychology*, *42*, 705-715.

Dumas, J. E., Nissley, J., Nordstrom, A., Smith, E. P., Prinz, R. J., & Levine, D. W. (2005). Home chaos: Sociodemographic, parenting, interactional, and child correlates. *Journal of Clinical Child and Adolescent Psychology*, *34*(1), 93-104.

Flouri, E., & Midouhas, E. (2017). Environmental adversity and children’s early trajectories of problem behavior: The role of harsh parental discipline. *Journal of family psychology*, *31*(2), 234.

Goodnight, J. A., Lahey, B. B., Van Hulle, C. A., Rodgers, J. L., Rathouz, P. J., Waldman, I. D., & D'Onofrio, B. M. (2012). A quasi-experimental analysis of the influence of neighborhood disadvantage on child and adolescent conduct problems. *Journal of abnormal psychology*, *121*(1), 95.

Hendriks, A. M., Finkenauer, C., Nivard, M. G., Van Beijsterveldt, C. E. M., Plomin, R. J., Boomsma, D. I., & Bartels, M. (2020). Comparing the genetic architecture of childhood behavioral problems across socioeconomic strata in the Netherlands and the United Kingdom. *European child & adolescent psychiatry*, *29*, 353-362.

Letourneau, N. L., Duffett-Leger, L., Levac, L., Watson, B., & Young-Morris, C. (2013). Socioeconomic status and child development: A meta-analysis. *Journal of Emotional and Behavioral Disorders*, *21*(3), 211-224.

Marsh, S., Dobson, R., & Maddison, R. (2020). The relationship between household chaos and child, parent, and family outcomes: a systematic scoping review. *BMC public health*, *20*, 1-27.

Matheny Jr, A. P., Wachs, T. D., Ludwig, J. L., & Phillips, K. (1995). Bringing order out of chaos: Psychometric characteristics of the confusion, hubbub, and order scale. *Journal of applied developmental psychology*, *16*(3), 429-444.

Mills-Koonce, W. Roger, et al. "The interplay among socioeconomic status, household chaos, and parenting in the prediction of child conduct problems and callous–unemotional behaviors." *Development and psychopathology* 28.3 (2016): 757-771.

Mulder, J. D., & Hamaker, E. L. (2021). Three extensions of the random intercept cross-lagged panel model. *Structural Equation Modeling: A Multidisciplinary Journal*, *28*(4), 638-648.

Tucker, C. J., Sharp, E. H., Van Gundy, K. T., & Rebellon, C. (2018). Household chaos, hostile parenting, and adolescents’ well-being two years later. *Journal of Child and Family Studies*, *27*, 3701-3708.

Winkleby, M. A., Jatulis, D. E., Frank, E., & Fortmann, S. P. (1992). Socioeconomic status and health: how education, income, and occupation contribute to risk factors for cardiovascular disease. *American journal of public health*, *82*(6), 816-820.

**Appendix 5. Post-hoc models separating emotional and behavioural problems**

| Table S4. Emotional problems - parameter estimates for the three models | | | | | | |  |  |  |  |  |  |
| --- | --- | --- | --- | --- | --- | --- | --- | --- | --- | --- | --- | --- |
|  | CLPM | | | | MZD-CLPM | | | | RI-CLPM | | | |
| Parameter | Std beta | SE | lower CI | upper CI | Std beta | SE | lower CI | upper CI | Std beta | SE | lower CI | upper CI |
|  |  |  |  |  |  |  |  |  |  |  |  |  |
| *Cross-lagged effects* |  |  |  |  |  |  |  |  |  |  |  |  |
| MP9 -> EM12 | **0.06** | **0.03** | **0.00** | **0.12** | **0.08** | **0.05** | **-0.02** | **0.17** | -0.02 | 0.05 | -0.12 | 0.08 |
| EM9 -> MP12 | 0.04 | 0.01 | 0.02 | 0.07 | -0.01 | 0.02 | -0.05 | 0.03 | -0.05 | 0.03 | -0.10 | 0.01 |
| MP12 -> EM16 | 0.00 | 0.04 | -0.08 | 0.08 | -0.01 | 0.06 | -0.12 | 0.11 | **-0.10** | **0.07** | **-0.24** | **0.04** |
| EM12 -> MP16 | 0.00 | 0.02 | -0.05 | 0.04 | 0.02 | 0.03 | -0.04 | 0.08 | **-0.10** | **0.04** | **-0.18** | **-0.03** |
|  |  |  |  |  |  |  |  |  |  |  |  |  |
| *Contemporaneous associations* |  |  |  |  |  |  |  |  |  |  |  |  |
| EM9 <-> MP9 | 0.17 | 0.09 | -0.01 | 0.35 | 0.04 | 0.13 | -0.21 | 0.29 | 0.07 | 0.14 | -0.21 | 0.35 |
| EM12 <-> MP12 | 0.10 | 0.05 | 0.00 | 0.20 | **0.07** | **0.09** | **-0.10** | **0.24** | 0.01 | 0.10 | -0.18 | 0.21 |
| EM 16 <-> MP16 | 0.12 | 0.10 | -0.08 | 0.32 | **0.12** | **0.18** | **-0.23** | **0.46** | 0.05 | 0.12 | -0.18 | 0.28 |
|  |  |  |  |  |  |  |  |  |  |  |  |  |
| *Autoregressive effects* |  |  |  |  |  |  |  |  |  |  |  |  |
| MP9 -> MP12 | 0.33 | 0.02 | 0.29 | 0.38 | 0.05 | 0.03 | -0.01 | 0.11 | **0.24** | **0.05** | **0.14** | **0.33** |
| MP12->MP16 | 0.29 | 0.03 | 0.23 | 0.35 | 0.05 | 0.03 | -0.02 | 0.11 | **0.19** | **0.07** | **0.06** | **0.32** |
| EM9 ->EM12 | 0.39 | 0.02 | 0.35 | 0.43 | **0.16** | **0.03** | **0.10** | **0.22** | **0.22** | **0.05** | **0.13** | **0.31** |
| EM12-> EM16 | **0.40** | **0.03** | **0.34** | **0.45** | **0.26** | **0.05** | **0.17** | **0.35** | **0.18** | **0.07** | **0.04** | **0.31** |
| *Between-person effects* |  |  |  |  |  |  |  |  |  |  |  |  |
| MP <-> EM |  |  |  |  |  |  |  |  | **0.72** | **0.13** | **0.47** | **0.96** |

Estimates with a p<.05 are in bold.

| Table S5. Behavioural problems - parameter estimates for the three models | | | | | | |  |  |  |  |  |  |
| --- | --- | --- | --- | --- | --- | --- | --- | --- | --- | --- | --- | --- |
|  | CLPM | | | | MZD-CLPM | | | | RI-CLPM | | | |
| Parameter | Std beta | SE | lower CI | upper CI | Std beta | SE | lower CI | upper CI | Std beta | SE | lower CI | upper CI |
|  |  |  |  |  |  |  |  |  |  |  |  |  |
| *Cross-lagged effects* |  |  |  |  |  |  |  |  |  |  |  |  |
| MP9 -> BP12 | **0.11** | **0.02** | **0.06** | 0.15 | -0.03 | 0.04 | -0.10 | 0.05 | **0.07** | **0.03** | **0.00** | **0.14** |
| BP9 -> MP12 | **0.15** | **0.02** | **0.11** | **0.19** | **0.08** | **0.03** | **0.03** | **0.13** | **0.10** | **0.03** | **0.04** | **0.16** |
| MP12 -> BP16 | **0.05** | **0.02** | **0.01** | **0.10** | 0.04 | 0.04 | -0.03 | 0.11 | -0.03 | 0.05 | -0.12 | 0.06 |
| BP12 -> MP16 | 0.06 | 0.03 | -0.01 | 0.12 | 0.02 | 0.05 | -0.08 | 0.11 | 0.00 | 0.05 | -0.10 | 0.09 |
|  |  |  |  |  |  |  |  |  |  |  |  |  |
| *Contemporaneous associations* | |  |  |  |  |  |  |  |  |  |  |  |
| BP9 <-> MP9 | **0.32** | **0.07** | **0.18** | **0.47** | **0.13** | **0.10** | **-0.06** | **0.33** | **0.26** | **0.10** | **0.07** | **0.45** |
| BP12 <-> MP12 | **0.20** | **0.04** | **0.11** | **0.28** | **0.10** | **0.06** | **-0.02** | **0.22** | **0.16** | **0.06** | **0.04** | **0.29** |
| BP 16 <-> MP16 | **0.16** | **0.06** | **0.04** | **0.28** | 0.06 | 0.10 | -0.14 | 0.25 | **0.10** | **0.08** | **-0.05** | **0.24** |
|  |  |  |  |  |  |  |  |  |  |  |  |  |
| *Autoregressive effects* |  |  |  |  |  |  |  |  |  |  |  |  |
| MP9 -> MP12 | **0.30** | **0.02** | **0.25** | **0.34** | 0.04 | 0.03 | -0.03 | 0.11 | **0.23** | **0.05** | **0.13** | **0.32** |
| MP12->MP16 | **0.28** | **0.03** | **0.21** | **0.34** | 0.05 | 0.05 | -0.05 | 0.15 | **0.20** | **0.07** | **0.07** | **0.33** |
| BP9 ->BP12 | **0.38** | **0.02** | **0.33** | **0.42** | **0.20** | **0.03** | **0.13** | **0.26** | **0.28** | **0.04** | **0.20** | **0.35** |
| BP12-> BP16 | **0.32** | **0.02** | 0.00 | 0.00 | **0.20** | **0.03** | **0.13** | **0.26** | **0.14** | **0.05** | **0.04** | **0.23** |
| *Between-person effects* |  |  |  |  |  |  |  |  |  |  |  |  |
| MP <-> BP |  |  |  |  |  |  |  |  | **0.76** | **0.09** | **0.59** | **0.93** |
| Estimates with a p<.05 are in bold. | | | | | | |  |  |  |  |  |  |

**Appendix 6. factorial measurement invariance of the RI-CLPM**

Upon reviewers request we have tested factorial measurement invariance of the R-CLPM. The code and results can be found here: <https://osf.io/jcu3h>.

A change in CFI smaller than .01 is an indication of measurement invariance supplemented by a change of ≥ - .015 in RMSEA , which is known as a reliable criterion for measurement invariance model comparisons (Chen, 2007; Cheung & Rensvold, 2002). The chi-square difference test was not used, because it is overly sensitive to trivial deviations in large samples (Marsh, Hau, & Grayson, 2005; Putnick & Bornstein, 2016). As displayed in Table S6, results from the model comparison showed that the factor loadings cannot be constrained over time, making further comparisons between the latent variables problematic. Because Weak Factorial Invariance was not reached we did not test further for Strong Factorial Invariance.

Table S6. Factorial Measurement Invariance

| RI-CLPM | χ² | *df* | CFI | TLI | RMSEA | SRMR | ΔCFI | ΔRMSEA |
| --- | --- | --- | --- | --- | --- | --- | --- | --- |
|  |  |  |  |  |  |  |  |  |
| Step 1: Configural Invariance | 1814.9 | 687 | 0.942 | 0.928 | 0.018 | 0.031 | - | - |
| Step 2: Weak Factorial Invariance | 2169.9 | 708 | 0.925 | 0.909 | 0.020 | 0.038 | 0.017 | -0.002 |

**References**

Chen, F. F. (2007). Sensitivity of goodness of fit indexes to lack of measurement invariance. *Structural equation modeling: a multidisciplinary journal*, *14*(3), 464-504.

Cheung, G. W., & Rensvold, R. B. (2002). Evaluating goodness-of-fit indexes for testing measurement invariance. *Structural equation modeling*, *9*(2), 233-255.

Marsh, H. W., Hau, K. T., & Grayson, D. (2005). Goodness of fit in structural equation models.

Putnick, D. L., & Bornstein, M. H. (2016). Measurement invariance conventions and reporting: The state of the art and future directions for psychological research. *Developmental review*, *41*, 71-90.

**Appendix 7. Histograms of maladaptive parenting items and inter-item correlations**

Upon reviewers request we have created histograms and calculated inter-item correlations of maladaptive parenting items.

Figure S2. Histograms of maladaptive parenting items at age 9, 12 and 16


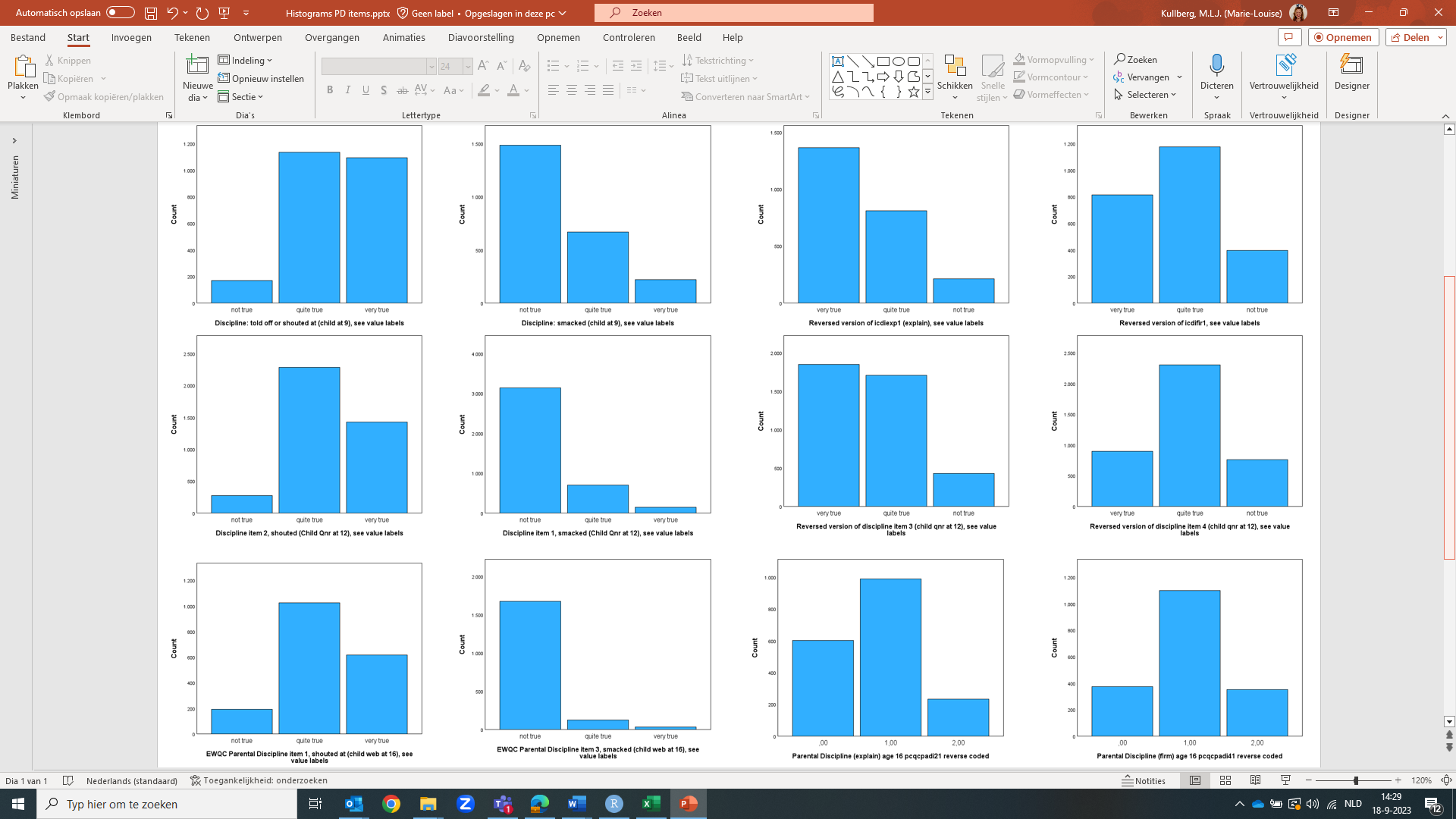


Table S7. Spearman’s Rho correlations between maladaptive parenting items

|  | Discipline: smacked (child at 9) | Discipline: told off or shouted at (child at 9) | Reversed version of explain (child at 9) | Reversed version of firm and calm (child at 9) | Discipline: smacked (child at 12) | Discipline: told off or shouted at (child at 12) | Reversed version of explain (child at 12) | Reversed version of firm and calm (child at 12) | Reversed version of explain (child at 16) | Reversed version of firm and calm (child at 16) | Discipline: told off or shouted at (child at 16) | Discipline: smacked (child at 16) |
| --- | --- | --- | --- | --- | --- | --- | --- | --- | --- | --- | --- | --- |
| Discipline: smacked (child at 9) | 1.000 |  |  |  | .349** | .128** | .046* | .141** | .020 | .062* | .090** | .174** |
| Discipline: told off or shouted at (child at 9) | .255** | 1.000 |  | .211** | .094** | .152** | .033 | .141** | .014 | .131** | .145** | .057* |
| Reversed version of explain (child at 9) | .045* | .002 | 1.000 | .272** | .026 | .012 | .209** | .092** | .118** | .033 | -.023 | -.014 |
| Reversed version of firm and calm (child at 9) | .200** | .211** | .272** | 1.000 | .119** | .061** | .140** | .175** | .080* | .131** | .040 | .078** |
| Discipline: smacked (child at 12) | .349** | .094** | .026 | .119** | 1.000 | .195** | .053** | .188** | .100** | .188** | .124** | .283** |
| Discipline: told off or shouted at (child at 12) | .128** | .152** | .012 | .061** | .195** | 1.000 | .029 | .143** | .006 | .163** | .134** | .112** |
| Reversed version of explain (child at 12) | .046* | .033 | .209** | .140** | .053** | .029 | 1.000 | .289** | .300** | .181** | -.055* | .043 |
| Reversed version of firm and calm (child at 12) | .141** | .141** | .092** | .175** | .188** | .143** | .289** | 1.000 | .174** | .257** | .038 | .081** |
| Reversed version of explain (child at 16) | .020 | -.003 | .069** | .070* | .065* | .037 | .232** | .124** | 1.000 | .318** | .174** | .123** |
| Reversed version of firm and calm (child at 16) | .062* | .083** | .021 | .081** | .124** | .116** | .118** | .167** | .318** | 1.000 | .187** | .223** |
| Discipline: told off or shouted at (child at 16) | .090** | .145** | -.023 | .040 | .124** | .134** | -.055* | .038 | .216** | .283** | 1.000 | .172** |
| Discipline: smacked (child at 16) | .174** | .057* | -.014 | .078** | .283** | .112** | .043 | .081** | .200** | .311** | .172** | 1.000 |
| ** Correlation is significant at the 0.01 level (2-tailed). | |  |  |  |  |  |  |  |  |  |  |  |
| * Correlation is significant at the 0.05 level (2-tailed). | |  |  |  |  |  |  |  |  |  |  |  |
